# Supplementary material for: Identification of photoperiod-induced specific miRNAs in the adrenal glands of Sunite sheep (Ovis aries)
Source: Front Vet Sci. 2022 Jul 22;9:888207. doi: 10.3389/fvets.2022.888207 (PMC9354845; doi:10.3389/fvets.2022.888207)
Supplement: Supplementary file 1 [file Data_Sheet_1.ZIP › Supplementary file/miRDeep2 file.zip!/miRDeep2 file/Know_Structure/SPLP42A3/oar-mir-1185.pdf]

miRBase precursor : oar-mir-1185
Total read count : 139
oar-miR-1185-5p read count : 39
oar-miR-1185-3p read count : 100
remaining reads : 0

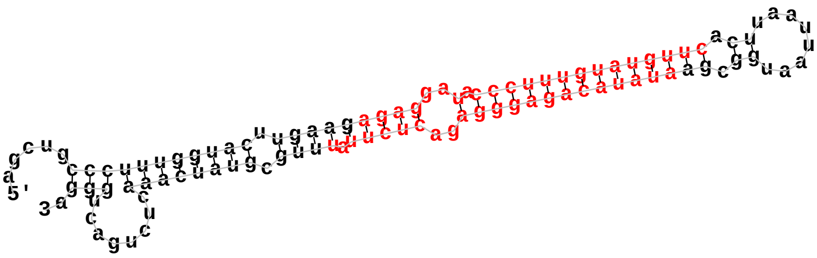

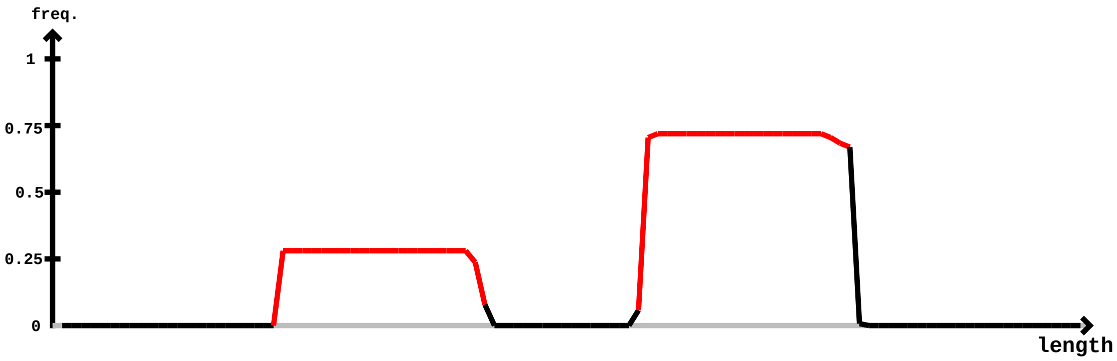


```
                                    oar-miR-1185-5p
                                                                                 oar-miR-1185-3p
5'- agcugcccuuugguacuugaagagaggauacccuuuguauguucacuuaauuaauggcgaauauacagagggagacucuuauuugcguaucaaacucugacuggga   -3'    exp
    .....(((((((((((.(((((((((..(.((((((((((((((.((........)).)))))))))))))))..))))).)))).))))))))........))).    reads    mm    sample
    .....................agaggauacccuuuguaugu.................................................................    5        0     seq
    .....................Ggaggauacccuuuguaugu.................................................................    1        1     seq
    .....................agaggauacccuuuguauguu................................................................    22       0     seq
    .....................agaggauacccuuuguauguuc...............................................................    11       0     seq
    ...........................................................aauauacagagggagacucu...........................    2        0     seq
    ...........................................................aauauacagagggagacucuuau........................    6        0     seq
    ............................................................auauacagagggagacucuu..........................    3        0     seq
    ............................................................auauacagagggagacucuua.........................    2        0     seq
    ............................................................auauacagagggagacucuuau........................    85       0     seq
    .............................................................uauacagagggagacucuuau........................    1        0     seq
    .............................................................uauacagagggagacucuuauu.......................    1        0     seq
```
